# Application of imaging mass spectrometry for the molecular diagnosis of human breast tumors

**DOI:** 10.1038/srep21043

**Published:** 2016-02-12

**Authors:** Xinxin Mao, Jiuming He, Tiegang Li, Zhaohui Lu, Jian Sun, Yunxiao Meng, Zeper Abliz, Jie Chen

**Affiliations:** 1 Department of Pathology, Peking Union Medical College Hospital, Chinese Academy of Medical Sciences and Peking Union Medical College, Beijing 100730, China; 2 State Key Laboratory of Bioactive Substance and Function of Natural Medicines, Institute of Materia Medica, Chinese Academy of Medical Sciences and Peking Union Medical College, Beijing 100050, China

## Abstract

Distinguishing breast invasive ductal carcinoma (IDC) and breast ductal carcinoma *in situ* (DCIS) is a key step in breast surgery, especially to determine whether DCIS is associated with tumor cell micro-invasion. However, there is currently no reliable method to obtain molecular information for breast tumor analysis during surgery. Here, we present a novel air flow-assisted ionization (AFAI) mass spectrometry imaging method that can be used in ambient environments to differentiate breast cancer by analyzing lipids. In this study, we demonstrate that various subtypes and histological grades of IDC and DCIS can be discriminated using AFAI-MSI: phospholipids were more abundant in IDC than in DCIS, whereas fatty acids were more abundant in DCIS than in IDC. The classification of specimens in the subtype and grade validation sets showed 100% and 78.6% agreement with the histopathological diagnosis, respectively. Our work shows the rapid classification of breast cancer utilizing AFAI-MSI. This work suggests that this method could be developed to provide surgeons with nearly real-time information to guide surgical resections.

Breast cancer (BC) is a complex and heterogeneous disease that has distinct biological features and clinical behaviors^1^,^2^. Tumor type^3^ and tumor grade^4^ are two of the most important characteristics, and they are the best-established prognostic factors in breast cancer. Among breast cancers, invasive ductal carcinoma (IDC) and breast ductal carcinoma *in situ* (DCIS) primarily occur in the extremities. IDC always requires radical treatment^5^, chemotherapy^6^,^7^ and radiotherapy^8^,^9^, but conservative treatment is usually sufficient for DCIS^10^,^11^. Currently, the differential diagnosis of DCIS and IDC can only be achieved after careful assessment (including histological and immunohistochemical assays) of the whole surgical specimen. Therefore, there is a critical need for precise molecular information to differentiate between DCIS and IDC during surgery. Such information could contribute to preventing the need for a second operation and in evaluating tumor cell invasion. Furthermore, numerous studies have demonstrated a significant association between tumor grade and breast cancer patient survival^12^. The prognosis for low-grade tumors is quite good, but high-grade tumors are more prone to distant metastasis and lead to a poor prognosis. Combining tumor type and tumor grade will allow a more accurate diagnosis and be more useful in guiding surgical resection^13^.

A variety of techniques have been developed to provide surgeons with information about breast cancer, including mammography^14^,^15^, ultrasound^16^,^17^, and magnetic resonance imaging (MRI)^7^. However, none of these offers the molecular information required to distinguish IDC from DCIS or evaluate the extent of tumor cell micro-invasion, especially during real-time imaging in a surgical environment. Clinical histopathology is the gold standard for clinical diagnosis, but many surgical tissues cannot be accurately diagnosed based solely on histomorphology, particularly in frozen tissue diagnosis because ice crystals lead to poor final slide quality and pathologic samplings in frozen are limited^18^.

AFAI-MSI is a novel mass spectrometry imaging method and represents a powerful tool for characterizing the lipidosis of biological tissues. In this approach, tissue imaging is not difficult to implement and does not require sample pretreatment or labeling. AFAI-MSI can produce a multicolor map to illustrate a tissue’s spatial distribution and the relative intensities of the molecules of interest. This technology produces reliable images of the spatial distribution of multiple molecules within a given section. The most important advantage of AFAI-MSI is the preservation of molecular characteristics, which could be used to observe the heterogeneous distribution of lipids in tissue sections. Additionally, AFAI-MSI is a rapid and nearly real-time analysis, and a single AFAI-MSI analysis of a tissue section requires only tens of minutes. Residue detection on a finger^19^ and whole-body molecular imaging^20^ have been achieved by AFAI-MSI. These results suggest that AFAI-MSI is a potential tool for in-procedure surgical resections.

## Result

A complete description of the samples used in the present study is provided in Supplementary Table S1. First, we developed a classification model that could distinguish the two types of breast cancer: the subtype classification, in which two groups were defined as 21 IDC samples and 19 DCIS samples; an independent cohort of 10 breast samples was used for validation. For the grade classification, the training cohort included 11 IDC samples and 11 DCIS samples of various grades, and the validation cohort included 28 breast cancer samples. Four distinct classifiers, including subtype and grade classification, were constructed as follows: comparison of IDC and DCIS in positive mode and negative mode; high-, intermediate-, and low-grade IDC in positive mode; high-, intermediate-, and low-grade DCIS in negative mode. The multivariate statistical methods used here included PCA and OPLS-DA. We correlated our findings with histopathology, and the classification of specimens in the subtype and grade validation sets showed 100% and 78.6% agreement with the histopathological diagnosis, respectively.

### Lipid profiles facilitate differentiating breast cancer tissues

The tissues from a total of 51 subjects were analyzed using AFAI-MSI. The results are outlined using the data obtained in both the positive and negative ion modes. The most prevalent lipid species observed in the breast cancer tissues in the mass range of *m/z* 100 to 1,000 were saturated and unsaturated fatty acids (FAs), phosphatidylcholine (PC), phosphatidylethanolamine (PE), and sphingomyelin (SM). Lipid ion imaging of the 2 different breast cancer subtypes and samples of different grades within each breast cancer subtype was then performed.

Here, we first report the mass spectrometric profiles distinguishing breast malignant tumors and normal tissue. Representative mass spectrometric profiles collected in positive ion mode from (A) an IDC tumor section and (B) IDC para-carcinoma tissues are shown in Supplementary Fig. S1. The overall signal acquired for the IDC tumor tissue is more intense than that obtained for IDC para-carcinoma because of a higher total lipid content in the *m/z* range of 500–700. Supplementary Fig. S2A–C shows an IDC tumor section, IDC para-carcinoma tissues and a breast fibroadenoma tumor with corresponding optical images. From these three samples, the distribution of several detected lipid species [*m/z* 706.5, PC (30:0); *m/z* 718.6, PC(32:0) or PE(35:0); *m/z* 724.5, PE(34:1); *m/z* 730.5, PC(32:2)] was found to be homogeneous in the ion images, and all of the ions that could distinguish breast cancer tissue from normal breast tissue or benign breast tissue were determined by AFAI-MSI. All images are plotted using the same intensity scale. Overlaying the histological image of the lipid ions confirms that these lipids are abundant in the tumor area but absent in normal breast lobule tissue and benign tumor areas. This finding suggests that the overall difference in lipid accumulation between a benign tumor and normal breast lobule tissue is not substantial.

### Associations with tumor type and grade

Unsupervised PCA was initially performed to obtain an overview of breast invasive ductal carcinoma or breast ductal carcinoma *in situ*, but separation trends between IDC and DCIS are not very obvious (Fig. S4 A and B). OPLS-DA was applied for the exploration of the difference between IDC and DCIS. For the training set, score scatter plots for OPLS-DA models from both positive and negative ion modes showed clear differentiation of IDC and DCIS (Fig. S4 C and D). In positive mode, classification of the IDC and DCIS groups resulted in one predictive (tB_pB_) and two orthogonal (tB_oB_) (1+2) components with a cross-validated predictive ability, *Q*^*2*^(cum), of 57.2%. In addition, 43.4% of the variance in *R*^*2*^(X) accounted for 71.7% of the variance of *R*^*2*^(Y). In negative mode, a data set was produced with an R^2^(X) of 40.6%, an R^2^(Y) of 85.5%, and a Q^2^(cum) of 73.1% across one predictive and two orthogonal components. The most prevalent lipid species observed in the examination of the IDC tissues in the mass range of *m/z* 500–1,000 were PC, PE and SM, whereas DCIS showed a distinct profile of lipid species containing fatty acids, including oleic acid and octadecanoic acid.

**Figure 1:**
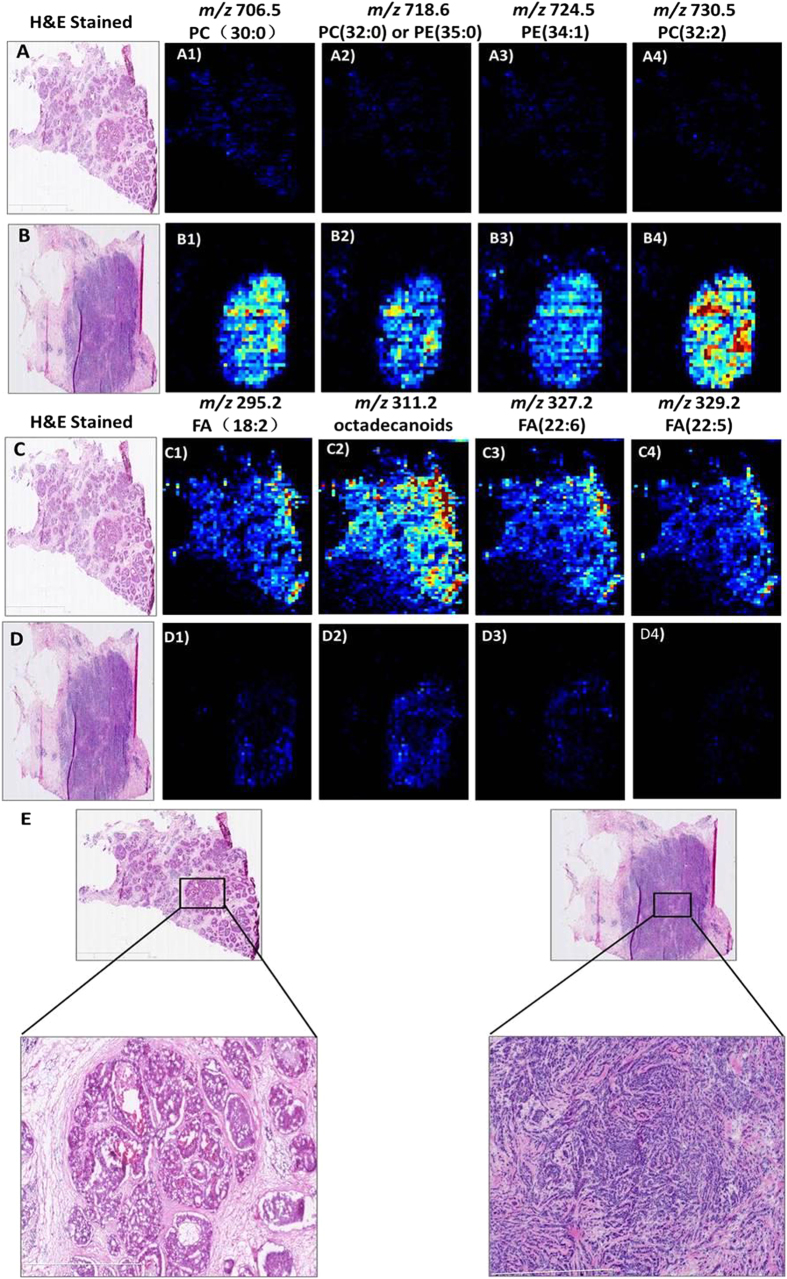
AFAI-MSI of samples of (**A,C**) low-grade breast ductal carcinoma *in situ* and (**B,D**) high-grade breast invasive ductal carcinoma. Positive ion mode AFAI-MSI ion images of sample N_49_ and sample N_22_ showing the distribution of (A1, B1) *m/z* 706.5, PC(30:0); (A2, B2) *m/z* 718.6, PC(32:0) or PE(35:0); (A3, B3) *m/z* 724.5, PE(34:1); and (A4, B4) *m/z* 730.5, PC(32:2). Negative ion mode AFAI-MSI ion images of sample N_49_ and sample N_22_ showing the distribution of (C1, D1) *m/z* 295.2, FA(18:2); (C2, D2) *m/z* 311.2, octadecanoids; (C3, D3) *m/z* 327.2, FA(22:6); and (C4, D4) *m/z* 329.2, FA(22:5). Lower magnification images with expanded views of an adjacent H&E-stained section are shown in (**E**).

**Figure 2:**
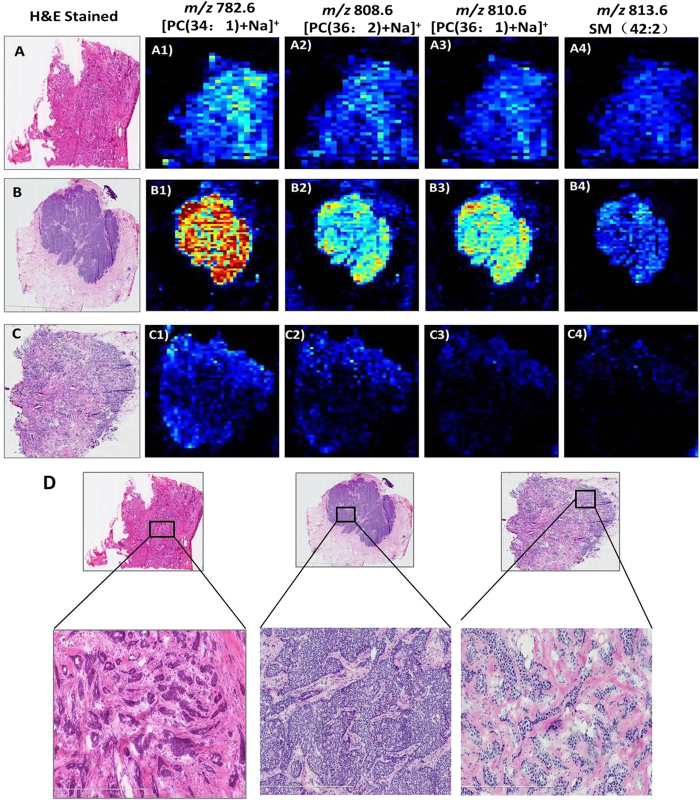
AFAI-MSI of (A1) low-, (B1) intermediate-, and (C1) high-grade breast invasive ductal carcinoma in samples N_5_, N_6_ and N_23_. Positive ion mode AFAI-MS ion images of (A1, B1, C1) *m/z* 782.6, [PC(34:1)+Na]^+^; (A2, B2, B3) *m/z* 808.6, [PC(36:2)+Na]^+^; (A3, B3, C3) *m/z* 810.6, [PC(36:1)+Na]^+^; and (A4, B4, C4) *m/z* 813.6, SM(42:2). Lower magnification images with expanded views of an adjacent H&E-stained section are shown in (**D**).

Figure 1A,B present the AFAI-MSI from the N49 and N22 samples. The ion maps of four representative ions [*m/z* 706.5, PC(30:0); *m/z* 718.6, PC(32:0) or PE(35:0); *m/z* 724.5, PE(34:1); and *m/z* 730.5, PC(32:2)] are present. These four ions are all abundant in IDC tumor tissue (Fig. 1B) but absent or weak in DCIS tumor tissue (Fig. 1A). Moreover, changes in the abundances of lipid species are observed as a function of grade for breast cancer, and such changes were found in low-, intermediate-, and high-grade IDC samples (Fig. 2). In the positive mode, low- and intermediate-grade (Fig. 2A,B) IDC samples showed the presence of PC and SM. Conversely, high-grade IDC (Fig. 2C), which expressed a more aggressive biological behavior, exhibited a lower total abundance of lipids than the low- and intermediate-grade IDC. For example, AFAI-MSI produced surgical N6 sample lipid ion maps that were characteristic of medial-grade breast cancer. Figure 2B shows that N6 is characterized by high relative abundances of the ions [*m/z* 782.6, [PC(34:1)+Na]^+^; *m/z* 808.6, [PC(36:2)+Na]^+^; *m/z* 810.6, [PC(36:1)+Na]^+^; and *m/z* 813.6, SM(42:2)], which is in agreement with the histopathological evaluation of an H&E-stained serial tissue section, this sample was diagnosed as medium-grade IDC.

**Figure 3:**
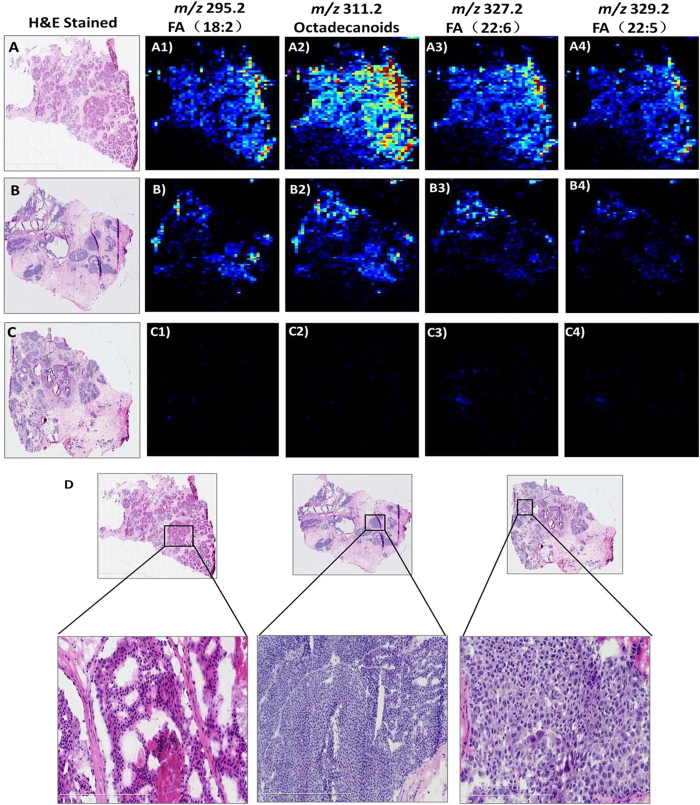
AFAI-MSI of breast cancer samples of (A1) low-, (B1) intermediate-, and (C1) high-grade breast ductal carcinoma *in situ* in samples N_49_, N_39_ and N_29_. Negative ion mode AFAI-MS ion images of (A1, B1, C1) *m/z* 295.2, FA(18:2); (A2, B2, C2) *m/z* 311.2, octadecanoids; (A3, B3, C3) *m/z* 327.2, FA(22:6); and (A4, B4, C4) *m/z* 329.2, FA(22:5). Lower magnification images with expanded views of an adjacent H&E-stained section are shown in (**D**).

**Figure 4:**
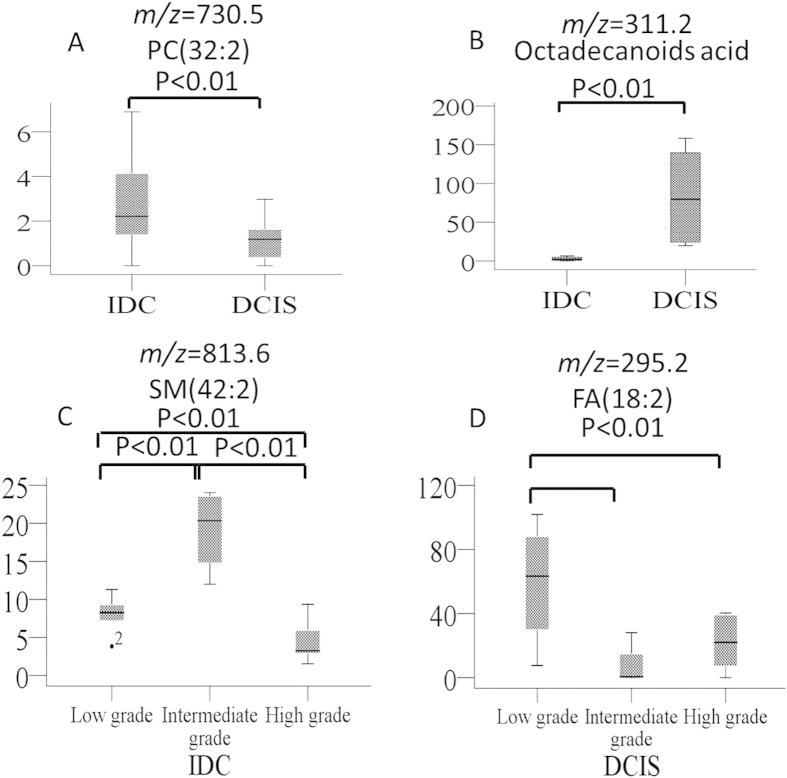
The statistical box plots show the ion intensity of (**A, B**) IDC and DCIS; (**C**) low-, intermediate-, and high-grade IDC; (**D**) low-, intermediate-, and high-grade DCIS.

The most abundant lipid species observed in DCIS were fatty acids, as shown in Fig. 1C,D: *m/z* 295.2, FA(18:2); *m/z* 311.2, octadecanoids; *m/z* 327.2, FA(22:6); and *m/z* 329.2, FA(22:5). These ions are abundant in the DCIS tumor areas (Fig. 1C) but weak or absent in regions corresponding to IDC tumor areas (Fig. 1D). Moreover, because no substantial differences were observed between high- and intermediate-grade DCIS tumors, we combined the high- and intermediate-grade tumors into one group and compared this group with the low-grade tumors. The fatty acid content in low-grade DCIS tumors (Fig. 3A) was substantially higher than that in the intermediate and high-grade DCIS tumors (Fig. 3B,C). These results were confirmed using individual candidate metabolites (Fig. 4). The median intensities of the characteristic peaks were statistically significant between distinct tissue types (P < 0.01)

To evaluate the predictive ability of the DCIS vs. IDC model, an external test was performed using 10 breast cancer samples from five specimens of DCIS and five specimens of IDC. None of these samples were included in the supervised analysis to ensure that the true predictive accuracy was determined. Satisfactory results were obtained. The two models (in positive and negative ion modes) achieved a predictive accuracy of 100%. Moreover, to further evaluate the predictive ability of high-, intermediate- and low-grade DCIS and high-, intermediate- and low-grade IDC tumor models, an independent test that included a set of 28 breast cancer samples was used. Only 6 differing results were noted for the specimens in the validation set, and the accuracy was 78.6%. These results show the substantial potential utility of AFAI-MSI analysis as a diagnostic technique in breast cancer.

**Figure 5:**
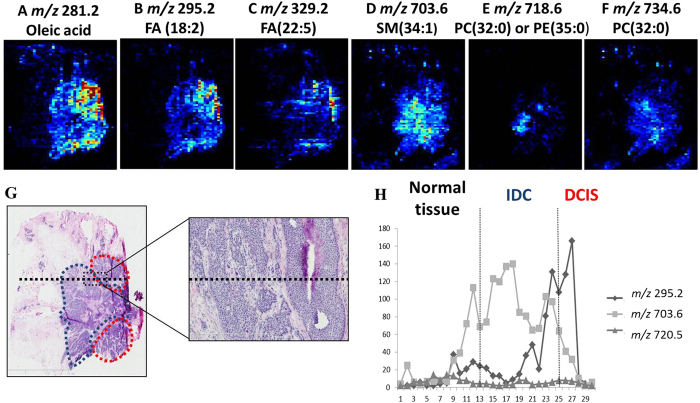
Tumor heterogeneity was assessed based on AFAI-MSI ion images of sample N_48_. Positive and negative ion images of (**A**) *m/z* 281.2, oleic acid; (**B**) *m/z* 295.2, FA(18:2); (**C**) *m/z* 329.2, FA(22:5); (**D**) *m/z* 703.6, SM(34:1); (**E**) *m/z* 718.6, PC(32:0) or PE(35:0); and (**F**) *m/z* 734.6, PC(32:0). An optical image of an adjacent H&E-stained section is shown in (**G**), with the region of breast invasive ductal cancer delineated with a dotted blue line, the region of breast ductal carcinoma *in situ* delineated by a dotted red line and a magnified border between these two regions. (**H**) plots the total abundance of the ions *m/z* 295.24, *m/z* 703.59, and *m/z* 720.54 by the distance (mm) along the dotted black line marked.

In some cases, DCIS occurs in combination with invasive cancer. A relatively homogeneous distribution of detected lipid species was found in the ion images of these cases. For example, surgical sample N48 (Fig. 5) showed marked heterogeneity according to AFAI-MSI. Two distinct regions were observed in the AFAI-MSI ion images obtained from sample. We not only found *m/z* 700–900 phospholipid profiles but also noted abundant free fatty acids (*m/z* 100–500) in this case. The tissue expressions of the two regions were then evaluated in the training and validation cohorts using phospholipids and fatty acids. This characteristic profile was observed for all samples harboring coexistent DCIS and IDC, demonstrating the capability of AFAI-MSI to distinguish between these cases. Two regions with distinctive lipid profiles were found in this tissue section (Fig. 5H). A detailed histopathological evaluation of an adjacent H&E-stained tissue section revealed that the region delineated in blue is IDC, whereas the region delineated in red is DCIS (Fig. 5G). The lipid profile in the IDC region was consistent with the characteristic lipid profile of other IDC samples, whereas a distinctly different lipid profile was detected in the DCIS region, which was consistent with the characteristic lipid profile observed for other DCIS samples (Fig. 5A–F). A similar significant difference was found for sample N24 in the validation cohorts for both phospholipids and fatty acids (Supplementary Fig. S3). The levels of phospholipids and fatty acids were higher in tumor cells than in normal breast lobular cells in both the training and validation cohorts. The utility of AFAI-MSI was demonstrated by its reliability in distinguishing between these 2 distinct tumor types based on lipid composition.

### Mass spectrometry imaging for micro-invasive breast cancer *in situ* by AFAI-MSI

**Figure 6:**
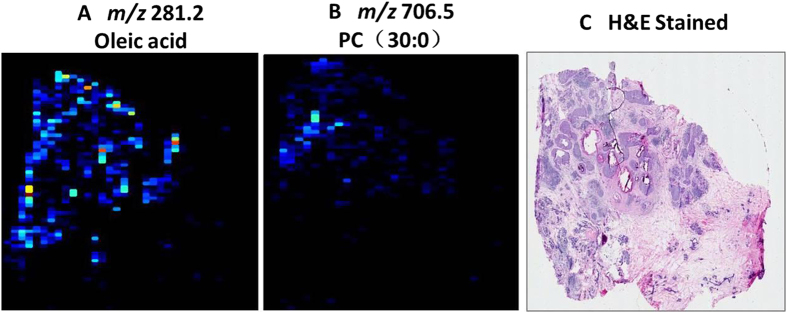
AFAI-MSI of breast ductal carcinoma *in situ* with focal micro-invasion. Positive and negative ion mode AFAI-MS ion images of sample N_29_ showing the distribution of (**A**) m/z 281.2, oleic acid; and (**B**) *m/z* 706.5, PC(30:0). An optical image of an adjacent H&E-stained section is shown in (**C**).

New diagnostic markers for micro-invasive breast carcinoma could have a major impact on clinical surgical removal. Determining the presence of micro-invasive cancer can be extremely difficult, particularly in high-grade ductal carcinoma *in situ* lesions. To assess whether phospholipids could be employed to detect micro-invasive cancer, we investigated cases of ductal carcinoma *in situ* with micro-invasive cancer. We found that these markers were also expressed in micro-invasive tumor cells, suggesting that they could be utilized to predict such cancers (Fig. 6). These markers could be beneficial for the selection of patients for clinical therapy at an early stage of aggressive breast cancer.

## Discussion

Mass spectrometry imaging (MSI) has been recognized as a powerful tool for the molecular imaging of tumors, including breast cancer. Matrix assisted laser desorption/ionization mass spectrometry imaging (MALDI MSI) has been used to study proteomic differences in breast cancer-associated stroma^21^ and in HER2 receptor status cases of breast cancer^22^. Desorption electrospray ionization (DESI) coupled with MSI has also already been shown to distinguish cancerous breast tissue from noncancerous tissue^23^; in addition, this approach is useful for tissue-specific metabolomic profiling^24^ and for the determination of tumor heterogeneity^25^,^26^. To our knowledge, this study is the first to demonstrate the use of MSI in the discrimination of IDC and DCIS and in the evaluation of tumor micro-invasion. Tumor infiltration is an important prognostic factor in breast cancer. Failure to discern the infiltrating tumor cells will increase the likelihood of requiring a second operation^27^.

In the present study, AFAI-MSI was not only applied to discriminate human breast cancer from adjacent normal breast tissue but also to compare the lipid compositions of various grades of IDC and DCIS in an attempt to systematically investigate the variation in molecular information in breast cancer subtypes and grades. Our study demonstrated that 23 lipids were differentially expressed in cases of IDC (Table 1) and that phospholipids were the most adversely affected, particularly in intermediate-grade tumors. In contrast, the AFAI-MSI spectra revealed higher levels of 6 fatty acid lipids in cases of DCIS, particularly in low-grade tumors (Table 1). Overall, our work provides a novel modality to integrate breast surgery and breast pathology to facilitate clinical decision making.

Only 6 discrepant results were found in the grade diagnosis validation set, which is a notably small number given the heterogeneity and complexity of breast cancers. Nearly all breast cancer grades exhibit diverse morphological characteristics. Differences in the classification results for the samples were observed mostly for IDC tumor grades, which showed differences between low grade and intermediate grade and between high grade and low grade, and DCIS tumor grade, which showed differences between low grade and intermediate grade and between high grade and intermediate grade (Table 2). These differences in grade between samples from the same surgical case were expected and reflect the heterogeneity in breast cancer. The chi-square test and Fisher’s exact test were used to verify whether or not the grade was statistically different between the histopathologic diagnosis and the observation by AFAI-MSI. No statistical significance was noted between the IDC group and the DCIS group (Table S2).

Lipids play important roles in maintaining cellular structure and forming membrane microdomains^28^, and a lipid imbalance in the membrane leads to changes in cell function and damage to growth mechanisms, which can lead to tumor formation. Accumulating evidence suggests that molecular changes in lipids are a widespread phenomenon in malignant tumor cells^29^,^30^. Tumor cells require increased metabolism to support tumor growth and proliferation, and cancer cells readapt their metabolic pathways to respond to these demands. As a result of this elevation, tumor cells could generate sufficient lipids to maintain lipid supplies and produce lipid precursors. Phospholipids are known to be involved in cell division^31^ and proliferation^32^,^33^, and additionally, they function as signaling molecules and second messengers^34^,^35^. PC serves as a direct substrate for SM synthesis in phospholipids. PC is also a major source of the second messenger DAG, phosphatidic acid and other signaling molecules. Moreover, PC is a mitogen for growth-factor induced DNA synthesis^36^,^37^. PE plays a major role in membrane architecture and can cause lateral membrane pressure^38^. PE is the precursor of many biologically active molecules, such as fatty acids and phosphatidic acid, which are all generated during PE metabolism^39^. PE plays an important role in cell division and ensures the proper progression of cytokinesis. Fatty acids can induce apoptosis in different cell types, and thus fatty acid synthase inhibitors may serve as anticancer targets^40^.

In our study, lipid and fatty acid levels increased in breast cancer, and DCIS had a higher concentration of fatty acids, whereas IDC had a higher level of phospholipids. These results suggest that the lipid changes observed in breast cancer could be related to pathways that are affected by tumor progression. These results are consistent with previous studies demonstrating that FAS expression and activity increase in human breast cancer cells and that these changes are unrelated to sex steroids or nutrition^41^,^42^. These lipids could be used for the molecular diagnosis of breast cancer; however, the underlying mechanisms have not yet been investigated. AFAI-MSI analysis of lipids in breast cancer revealed differences between low- and high-grade tumors. The spectra of high-grade tumors revealed lower levels of PC than in intermediate- and low-grade tumors. This result is not consistent with earlier studies by Mika Hilvo and coworkers^43^, who demonstrated that the highest levels of phospholipids were found in the most aggressive tumors (grade 3). Thus, our results challenge earlier proposals that the overexpression of FAS is most intense in carcinomas that carry higher risks.

In conclusion, we demonstrated that AFAI-MSI can be utilized to distinguish between different types and grades of breast cancer based on lipid signatures. Molecular information obtained using AFAI-MSI analysis can provide insight into the biochemical processes that occur during malignancy. Future studies of precancerous tissues, such as atypical hyperplasia, are currently under consideration as a possible extension of our work.

## Materials and Methods

### Sample collection

All tissue samples were breast tumor tissues collected at Peking Union Medical College Hospital between 2012 and 2013. Study protocols were approved by the Ethical Review Community of Peking Union Medical College Hospital. All patients involved consented to participate in the study and signed an informed consent form, and all experiments were performed in accordance with the approved guidelines. None of the 51 breast cancer cases received preoperative treatment. The tumor tissue samples obtained from surgical resection specimens were snap-frozen and stored at -80°C until sectioning. For the AFAI-MSI experiments, 8-μm-thick tissue sections were cut at −20 °C using a cryomicrotome (CM 1950; Leica, Wetzler, Germany) and thaw-mounted onto microscope glass slides (Superfrost Plus slides, Thermo Fisher Scientific, USA). The slides were stored in closed containers at −80 °C. Prior to analysis, the slides were allowed to thaw at room temperature and were dried in a desiccator for approximately 30 minutes. Five-micrometer-thick tissue sections were cut and stained with hematoxylin and eosin (H&E) to confirm the diagnosis.

### AFAI-MSI analysis

The mass spectrometry imaging experiments were performed using a Q-TOF (QSTAR Elite, Applied Biosystem/MDS Sciex) equipped with a custom-made AFAI ion source. Data were acquired in both the positive and negative ion modes. The measurement conditions were as follows: the ESI sprayer was positioned 0.7 mm away from the tissue surface at an incident angle of 55° to the sample surface. The spray solvent for MS acquisition in the positive mode was acetonitrile: water (8:2, v/v) containing 0.1% formic acid with a 4,600-V spray voltage. Acetonitrile (ultra-pure water) was purchased from Germany. The solvent flow rate was 5 μL/min, and the extraction flow rate was 45 L/min. In the tissue-imaging experiments, each tissue section was scanned using a 2D moving stage with horizontal rows separated by a 200-μm vertical step until the entire tissue sample was assayed. All experiments were conducted using Analyst QS 2.0 software. Mass spectra were rescored in the mass range of *m/z* 100–1000.

### Histopathology analysis

Tumor content was determined by 2 pathologists through the examination of the H&E-stained tissue section adjacent to the section used for the AFAI-MSI studies. The slides were re-evaluated histologically and classified according to the 2012 World Health Organization criteria. DCIS and IDC cases were histologically graded according to the Bloom-Richardson grading system. Tumor content was determined by 2 pathologists through the examination of the H&E-stained tissue section adjacent to the section used for the AFAI-MSI studies. The tissue slides were free of necrosis. The specimens were evaluated and scored visually with respect to tubule formation, nuclear pleomorphism and mitotic count.

### Data processing and statistical analysis

We divided all samples into training and validation sets for statistical analysis. All tissue samples were subjected to AFAI-MSI analysis. Raw data files acquired from AFAI-MS analysis were initially converted to Matlab format using Wiff-to-Matlab translator software. A software tool was developed to enable the visual inspection of the ion image and the selection of regions of interest (ROIs). The ROIs were selected via H&E staining, and the corresponding spectral data were extracted from the ROI. The resultant 2D matrices, including observations (sample names) in columns and variables (*m/z*) in rows, were subsequently transferred to Makerview (Applied Biosystems/MDS Sciex) and to the SIMCA-P 12.0 software package (Umetrics AB, Umeå, Sweden) to build classification models. Pareto scaling and normalization with ion intensity were applied to all data in order to reduce noise and artefacts in the models. The multivariate statistical methods used here included principal components analysis (PCA) and orthogonal partial least square discriminant analysis (OPLS-DA). Four distinct classifiers were built to identify breast cancer subtypes and grades. The training set models were used to differentiate between the statistical and validation sets. After classification, discriminating variables were selected according to variable importance (VIP) and assessed using an independent t-test (Microsoft Office Excel 2010). P < 0.05 was considered statistically significant.

### Molecular identification

Accurate mass measurement and MS/MS analysis were performed on a Q-Orbitrap mass spectrometer (Q-Exactive, Thermofisher) for molecular identification. Lipid species were first analyzed by comparing each peak’s mass measurement with that in the LIPID MAPS database (http://lipidmaps.org; mass accuracy ± 0.005 Da) and then identified based on collision-induced dissociation (CID) experiments, tandem MS experiments and comparison with data from the literature.

## Additional Information

**How to cite this article**: Mao, X. *et al.* Application of imaging mass spectrometry for the molecular diagnosis of human breast tumors. *Sci. Rep.* **6**, 21043; doi: 10.1038/srep21043 (2016).

## Supplementary Material

Supplementary Information

## Figures and Tables

**Table 1:** Peaks used to distinguish the breast cancer subtypes and grades with identification and MS fragment information.

Peak (*m/z*)	VIP	Identification	Main product Ions (*m/z*)	Peak(*m/z*)	VIP	Identification	Main product Ions (*m/z*)
Subtype							
703.6	6.5	SM(34:1)	184.0732	770.6	3.9	[PC(32:1)+K]^+^ or	711.4349,
							629.5487,
						[PE(35:1)+K]^+^	184.0731,
							162.9556
718.6	4.5	PC(32:0) or PE(35:0)	700.4153, 577.5189, 184.0733,	782.6	6.7	[PC(34:1)+Na]^+^	723.4922,
							599.5002,
							184.0731,
							146.9816
706.5	3.8	PC(30:0)	184.0732,	794.5	3.2	[PC(34:3)+K]^+^ or	735.4342,
							653.5486,
						[PE(37:3)+K]^+^	184.0731,
							162.9555,
							146.9816
724.5	4.6	PE(34:1)	583.5080	796.5	3.3	[PC(34:2)+K]^+^ or	737.4477, 655.5644,
						[PE(37:2)+K]^+^	613.4794,
							184.0731,
							162.9555,
							146.9816
730.5	4.1	PC(32:2)	184.0733,	810.6	6.5	[PC(36:1)+Na]^+^	751.5219,
							184.0731
732.6	10.8	PC(32:1)	184.0733,	281.2	5.2	Oleic acid	68.9,
							112.9
734.6	6.3	PC(32:0)	184.0733,	295.2	4.0	FA(18:2)	–
744.5	5.5	[PE(33:0)+Na]^+^ or	685.4192,	297.2	6.4	FA(18:0)	68.9,
		[PC(30:0)+K]^+^	603.5340,				61.9,
			184.0733				96.9
746.6	4.8	PC(O-34:1) or	605.5492,	311.2	5.5	Octadecanoids	61.9,
		PE(O-37:1)	184.0733				69.0,
							96.9
752.6	3.5	[PE(P-36:1)+Na]^+^	611.5387	327.2	3.8	FA(22:6)	–
754.5	3.1	[PC(32:1)+Na]^+^	695.4609,	329.2	3.0	FA(22:5)	–
			571.4688,				
			184.0732,				
			146.9816,				
756.6	4.2	[PC(32:0)+Na]^+^	697.4771,				
			573.4895,				
			551.5031,				
			184.0731,				
			146.9816				
758.6	13.1	PC(34:2)	699.4350,				
			184.0731,				
			162.9554				
760.6	16.7	PC(34:1)	184.0731				
	766.6	4.5	[PC(34:1)+Na]^+^ or	707.4965,				
			625.5157,					
		[PE(37:1)+Na]^+^	184.0731,					
			164.0081,					
		146.9816						
grade								
732.6	10.9	PC(32:1)	184.0733	295.2	4.0	FA(18:2)	–	
760.6	16.7	PC(34:1)	184.0731	297.2	6.4	FA(18:0)	68.9,	
							61.9,	
							96.9	
786.6	8.2	PC(36:2)	592.6743,	311.2	5.5	Octadecanoids acid	61.9,	
			552.6070,				69.0,	
		184.0731				96.9		
808.6	3.2	[PC(36:2)+Na]^+^	749.5081,	327.2	3.8	FA(22:6)	–	
			625.5156,					
			184.0731,					
			146.9816					
810.6	3.2	[PC(36:1)+Na]^+^	751.5219,	329.2	3.0	FA(22:5)	–	
			184.0731					
813.6	5.0	SM(42:2)	184.0731					

**Table 2:** Classification results for subtype and grade classifiers applied to the validation set of samples.

Sample	Histopathology Diagnosis	Subtype	Grade
N1	IDC, low grade	IDC	*High*
N2	IDC, low grade	IDC	low
N4	IDC, low grade	IDC	low
N5	IDC, low grade	IDC	low
N7	IDC, intermediate grade	IDC	intermediate
N8	IDC, intermediate grade	IDC	intermediate
N9	IDC, intermediate grade	IDC	intermediate
N10	IDC, intermediate grade	IDC	intermediate
N12	IDC, high grade	IDC	high
N13	IDC, high grade	IDC	*intermediate*
N17	IDC, high grade	IDC	high
N21	IDC, high grade	IDC	*intermediate*
N23	IDC, high grade	IDC	*intermediate*
N25	IDC, high grade	IDC	high
N27	DCIS, high grade	DCIS	*intermediate*
N33	DCIS, high grade	DCIS	high
N34	DCIS, high grade	DCIS	high
N35	DCIS, high grade	DCIS	high
N36	DCIS, high grade	DCIS	high
N38	DCIS, intermediate grade	DCIS	intermediate
N39	DCIS, intermediate grade	DCIS	intermediate
N41	DCIS, intermediate grade	DCIS	intermediate
N42	DCIS, intermediate grade	DCIS	intermediate
N44	DCIS, intermediate grade	DCIS	intermediate
N45	DCIS, low grade	DCIS	low
N46	DCIS, low grade	DCIS	low
N48	DCIS, low grade	DCIS	low
N49	DCIS, low grade	DCIS	*intermediate*

NOTE: Italicized results are in apparent disagreement with diagnosis.

Abbreviations: IDC = invasive ductal carcinoma; DCIS = breast ductal carcinoma *in situ*. According to 2012 World Health Organization criteria: IDC, low grade = IDC, grade 1; IDC, intermediate grade = IDC, grade 2; IDC, high grade = IDC, grade 3.
