# Supplementary material for: Application of imaging mass spectrometry for the molecular diagnosis of human breast tumors
Source: Sci Rep. 2016 Feb 12;6:21043. doi: 10.1038/srep21043 (PMC4751527; doi:10.1038/srep21043)
Supplement: Supplementary Information [file srep21043-s1.doc]

**Application of imaging mass spectrometry for the molecular diagnosis of human breast tumors**

Xinxin Mao^1┼^, Jiuming He^2┼^, Tiegang Li^2^, Zhaohui Lu^1^, Jian Sun^1^, Yunxiao Meng ^1^, Zeper Abliz^2*^, Jie Chen^1*^

1 Department of Pathology, Peking Union Medical College Hospital, Chinese Academy of Medical Sciences and Peking Union Medical College, Beijing 100730, China

2 State Key Laboratory of Bioactive Substance and Function of Natural Medicines, Institute of Materia Medica, Chinese Academy of Medical Sciences and Peking Union Medical College, Beijing 100050, China

^*^ Corresponding author: Prof. Dr. Jie Chen and Prof. Dr. Zeper Abliz

Address: No. 1 Shuaifu Yuan street, Dongcheng District, Beijing 100730, P. R. China, Email: xhblk@163.com (J. C.)

Address: No. 1 Xiannongtan street, Xicheng District, Bejing 100050, P. R. China, Email: zeper@imm.ac.cn (Z. A.)

**^┼^**Equal contributors

**Table S1. Summary of the human breast cancer** specimens used in this study.

| **Sample** | **Diagnosis** | **Age** | **Site** | **Maximum diameter** | **Lymph node metastasis** | **ER** | **PR** | **HER-2** |
| --- | --- | --- | --- | --- | --- | --- | --- | --- |
| N1 | IDC, low grade | 65 | L | 1.8 cm | N | Positive | Positive | Positive |
| N2 | IDC, low grade | 37 | L | 1.2 cm | Y | Positive | Positive | Negative |
| N3 | IDC, low grade | 53 | R | 1.4 cm | N | Positive | Positive | Negative |
| N4 | IDC, low grade | 51 | L | 1.7 cm | N | Positive | Positive | Negative |
| N5 | IDC, low grade | 53 | L | 1.5 cm | N | Positive | Positive | Negative |
| N6 | IDC, intermediate grade | 40 | L | 1.5 cm | N | Positive | Positive | Negative |
| N7 | IDC, intermediate grade | 45 | R | 2.8 cm | N | Positive | Positive | Positive |
| N8 | IDC, intermediate grade | 50 | R | 1.5 cm | N | Positive | Positive | Positive |
| N9 | IDC, intermediate grade | 52 | R | 1.5 cm | N | Positive | Positive | Negative |
| N10 | IDC, intermediate grade | 44 | L | 2.5 cm | Y | Positive | Positive | Negative |
| N11 | IDC, intermediate grade | 53 | L | 2 cm | N | Positive | Positive | Negative |
| N12 | IDC, high grade | 74 | R | 2.3 cm | N | Positive | Positive | Positive |
| N13 | IDC, high grade | 24 | R | 1.6 cm | Y | Positive | Positive | Positive |
| N14 | IDC，high grade | 49 | R | 2.0 cm | N | Positive | Positive | Negative |
| N15 | IDC, high grade | 41 | R | 2.2 cm | N | Positive | Positive | Positive |
| N16 | IDC, high grade | 53 | L | 2.5 cm | N | Positive | Positive | Negative |
| N17 | IDC, high grade | 56 | R | 1.5 cm | N | Positive | Positive | Positive |
| N18 | IDC, high grade | 73 | L | 2.1 cm | Y | Positive | Positive | Negative |
| N19 | IDC, high grade | 52 | L | 2.3 cm | N | Positive | Positive | Negative |
| N20 | IDC, high grade | 37 | L | 2.2 cm | Y | Positive | Positive | Positive |
| N21 | IDC, high grade | 50 | L | 3.2 cm | N | Positive | Positive | Positive |
| N22 | IDC, high grade | 42 | L | 1.7 cm | N | Positive | Positive | Negative |
| N23 | IDC, high grade | 83 | L | 1.7 cm | N | Positive | Positive | Negative |
| N24 | IDC, high grade; focal DCIS, low grade | 42 | R | 1.3 cm | N | Positive | Positive | Positive |
| N25 | IDC, high grade; focal DCIS, high grade | 54 | L | 1.8 cm | N | Positive | Positive | Negative |
| N26 | IDC, high grade | 74 | L | 4.0 cm | Y | Positive | Positive | Negative |
| N27 | DCIS, high grade and IDC, intermediate grade | 45 | R | 2.8 cm | N | Positive | Positive | Positive |
| N28 | DCIS, high grade; focal microinvasion | 40 | L | 2.5 cm | Y | Positive | Positive | Positive |
| N29 | DCIS, high grade; focal microinvasion | 51 | L | 1.1 cm | N | Positive | Positive | Positive |
| N30 | DCIS, high grade and IDC, intermediate grade | 84 | R | 1.0 cm | N | Positive | Positive | Negative |
| N31 | DCIS, high grade | 34 | L | 1.1 cm | N | Positive | Positive | Positive |
| N32 | DCIS, high grade | 40 | R | 6.0 cm | N | Positive | Positive | Negative |
| N33 | DCIS, high grade | 58 | R | 1.7 cm | N | Positive | Positive | Positive |
| N34 | DCIS, high grade | 50 | R | 2.2 cm | Y | Positive | Positive | Negative |
| N35 | DCIS, high grade | 50 | L | 2.2 cm | N | Positive | Positive | Positive |
| N36 | DCIS, high grade | 38 | L | 2.2 cm | N | Positive | Positive | Negative |
| N37 | DCIS, high grade | 44 | L | 1.0 cm | Y | Positive | Positive | Negative |
| N38 | DCIS, intermediate grade | 44 | L | 3.0 cm | N | Positive | Positive | Negative |
| N39 | DCIS, intermediate grade | 54 | L | 2.2 cm | N | Positive | Positive | Positive |
| N40 | DCIS, intermediate grade | 55 | R | 2.2 cm | Y | Positive | Positive | Negative |
| N41 | DCIS, intermediate grade | 36 | R | 2.0 cm | N | Positive | Positive | Negative |
| N42 | DCIS, intermediate grade | 45 | L | 2.2 cm | N | Positive | Positive | Negative |
| N43 | DCIS, intermediate grade | 34 | R | 3.1 cm | Y | Positive | Positive | Negative |
| N44 | DCIS, intermediate grade | 38 | R | 1.9 cm | N | Positive | Positive | Negative |
| N45 | DCIS, low grade and IDC, intermediate grade | 50 | R | 1.0 cm | N | Positive | Positive | Negative |
| N46 | DCIS, low grade | 73 | L | 1.3 cm | N | Positive | Positive | Negative |
| N47 | DCIS, low grade | 38 | R | 1.4 cm | N | Positive | Positive | Negative |
| N48 | DCIS, low grade; focal IDC, intermediate grade | 42 | R | 1.9 cm | N | Positive | Positive | Positive |
| N49 | DCIS, low grade | 45 | R | 1.3 cm | N | Positive | Positive | Negative |
| N50 | DCIS, low grade | 53 | L | 2.5 cm | Y | Positive | Positive | Negative |
| N51 | breast fibroadenoma | 51 | R | 3.9 cm | N | Positive | Positive | Negative |

**DCIS = breast ductal carcinoma in situ; IDC = invasive ductal carcinoma; L = left; R = right; Y = yes; N = no;** According to 2012 World Health Organization criteria，IDC, low grade= IDC，grade 1；IDC, intermediate grade= IDC, grade 2；IDC, high grade= IDC, grade 3


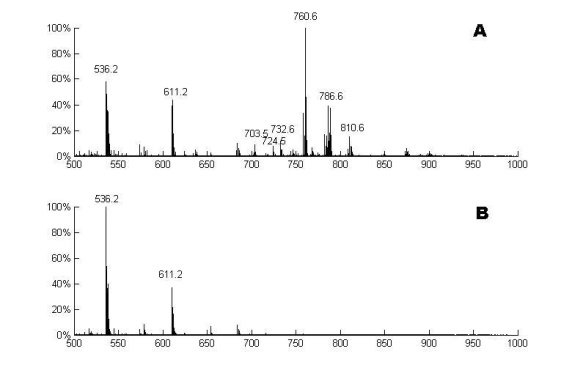


**Figure S1** Discrimination between normal and cancer tissue based on lipid profiles as detected by AFAI-MS. Representative positive ion mode mass spectra from breast cancer, (A) breast invasive ductal cancer (B) normal breast tissue.


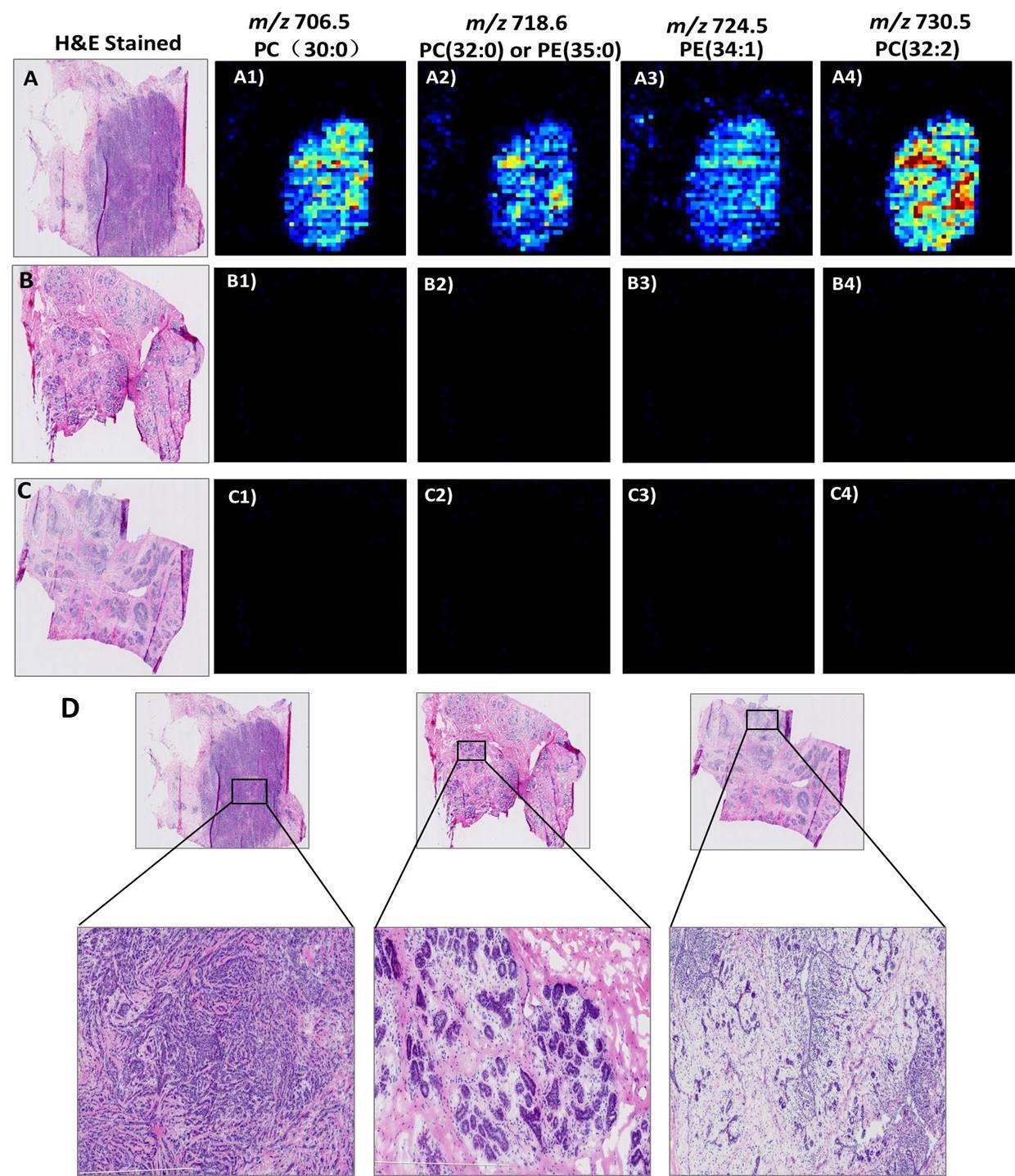


**Figure S2** AFAI-MSI of a (A) breast invasive ductal cancer sample, (B) para-carcinoma tissue and (C) fibroadenoma of breast tissue. Positive ion mode AFAI-MS ion images of sample N_22_ and sample N_51_ showing the distribution of (A1, B1, C1) *m/z* 706.5, PC (30:0); (A2, B2, C2) *m/z* 718.6, PC(32:0) or PE(35:0); (A3, B3, C3) *m/z* 724.5, PE(34:1); (A4, B4, C4) *m/z* 730.5, PC(32:2). Lower magnification images with expanded views of an adjacent H&E-stained section are shown in (D).


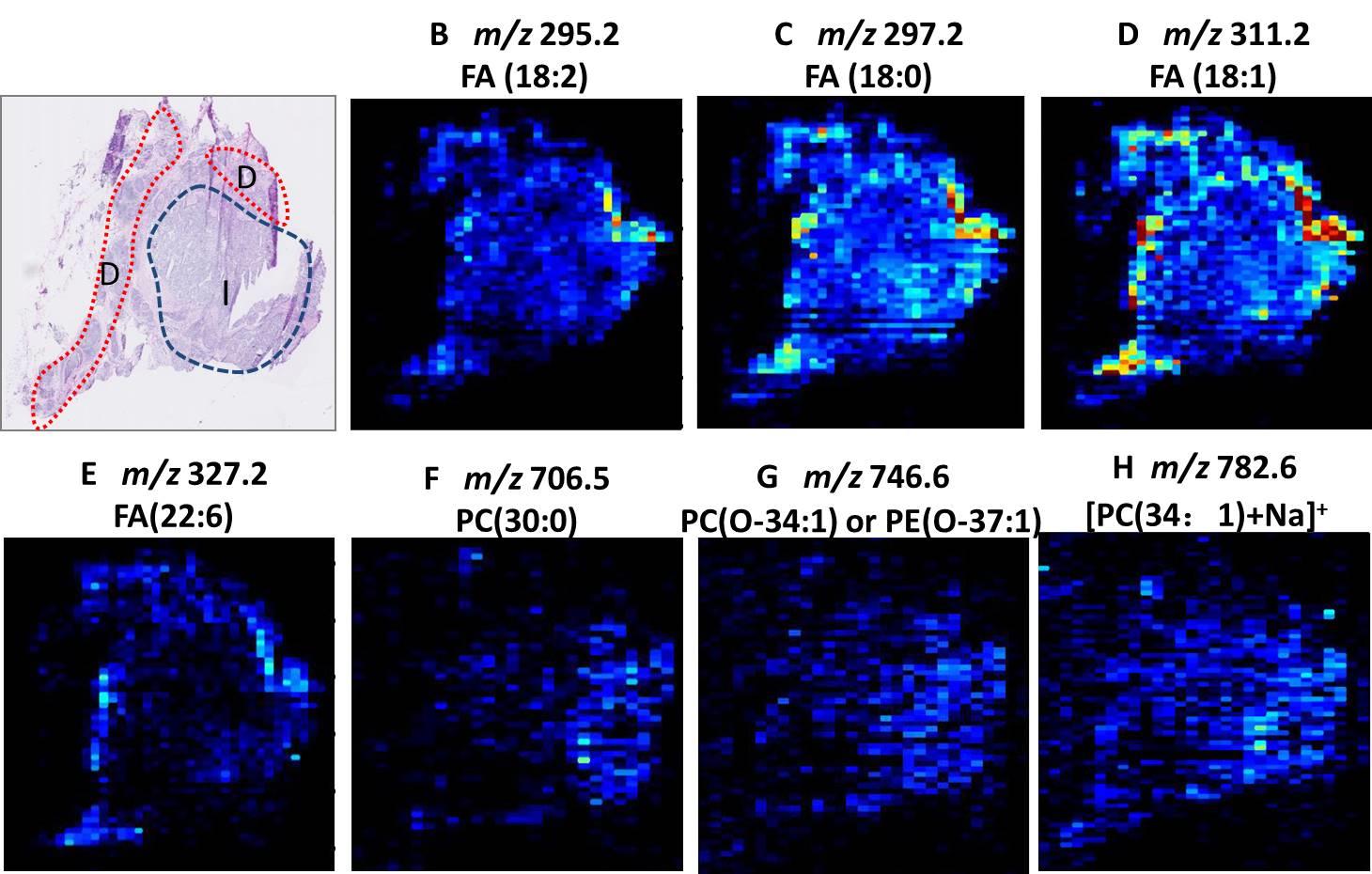


**Figure S3.** AFAI-MSI of a breast cancer sample. Positive and negative ion mode AFAI-MS ion images of sample N_27_ showing the distribution of (B) *m/z* 295.2, FA(18:2); (C) *m/z* 297.2, FA(18:0); (D) *m/z* 311.2, FA(18:1); (E) *m/z* 327.2, FA(22:6); (F) *m/z* 706.5, PC(30:0); (G) *m/z* 746.6, PC(O-34:1) or PE(O-37:1); and (H) *m/z* 782.6, [PC(34：1)+Na]^+^.


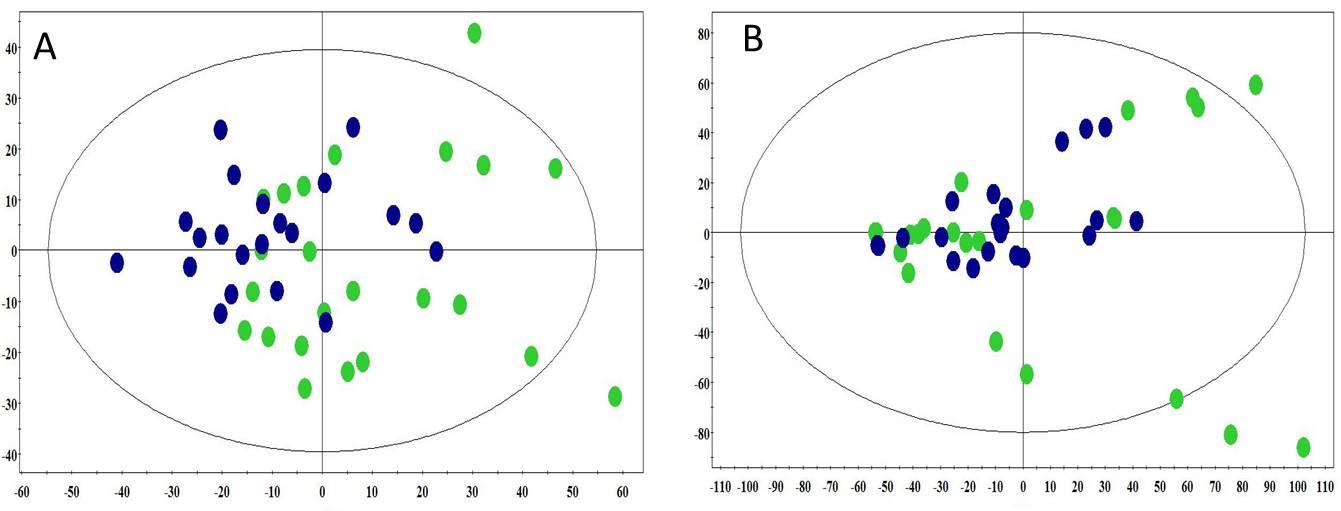


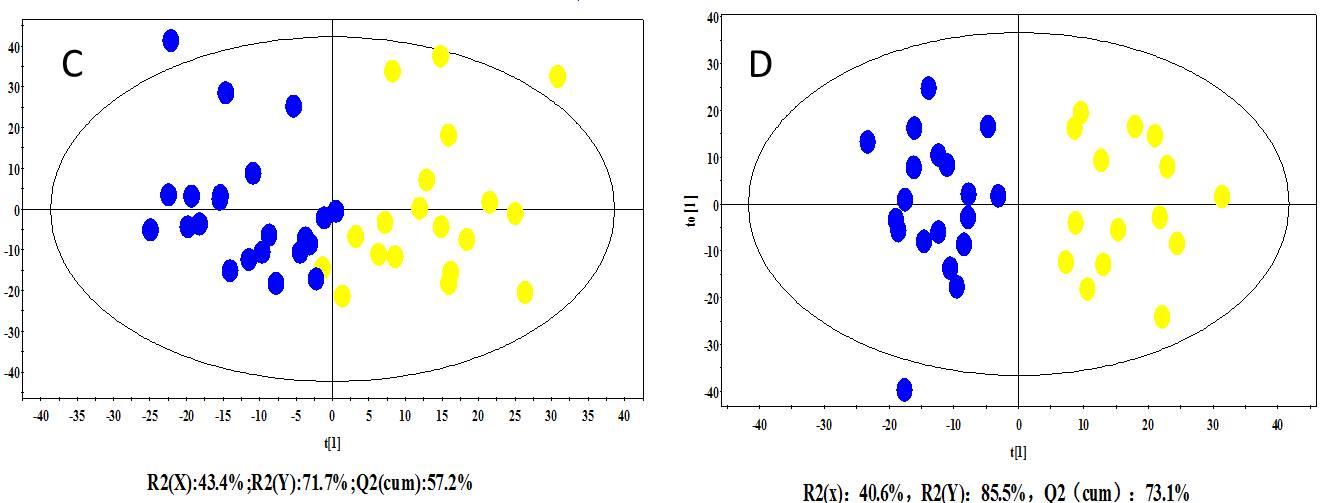


**Figure S4** PCA score plots of breast ductal invasive cancer vs. breast ductal carcinoma in situ (A) positive ion mode and (B) negative ion mode; Score plot of OPLS-DA of breast ductal invasive cancer vs. breast ductal in situ in positive ion mode (C) and negative ion mode(D).

**Table S2.** Statistical association between pathology diagnosis and the AFAI-MSI for IDC and DCIS various grade

| Pathology diagnosis | | | | | | |  |
| --- | --- | --- | --- | --- | --- | --- | --- |
| AFAI-MSI | IDC |  | low grade | intermediate grade | high grade | *P* value | *χ^2^* |
|  |  | low grade | 3 | 0 | 0 |  |  |
|  |  | intermediate grade | 0 | 4 | 3 | 10.399 | .026 |
|  |  |  |  |  |  |  |  |
|  |  | high grade | 1 | 0 | 3 |  |  |
|  | DCIS |  | high grade | intermediate grade | low grade |  |  |
|  |  | high grade | 4 | 0 | 0 |  |  |
|  |  | intermediate grade | 1 | 5 | 1 | 13.090 | .000 |
|  |  | low grade | 0 | 0 | 3 |  |  |

**IDC = invasive ductal carcinoma; DCIS = breast ductal carcinoma in situ;**

According to 2012 World Health Organization criteria: IDC, low grade= IDC，grade 1; IDC, intermediate grade= IDC, 2 grade; IDC, high grade= IDC, 3 grade.
